# Impact of Pregestational Weight and Weight Gain during Pregnancy on Long-Term Risk for Diseases

**DOI:** 10.1371/journal.pone.0168543

**Published:** 2017-01-03

**Authors:** Ulrika Moll, Håkan Olsson, Mona Landin-Olsson

**Affiliations:** 1 Department of Clinical Sciences, Lund University, Lund, Sweden; 2 Department of Endocrinology, Skane University Hospital, Lund, Sweden; 3 Department of Oncology, Skane University Hospital, Lund, Sweden; Centro Cardiologico Monzino, ITALY

## Abstract

**Objective:**

The aim of this study was to analyse the impact of maternal BMI at start of pregnancy and maternal weight gain during pregnancy on the risk of various diseases later in life.

**Methods:**

In a population-based cohort from southern Sweden, women with at least one delivery registered in the Swedish Medical Birth Register ten or more years before answering a health questionnaire were identified (n = 13,608). Complete data were found in 3,539 women.

**Results:**

Women with BMI >25 at start of pregnancy had increased risk of developing obesity (OR 21.9), diabetes (OR 6.4), cardiac disease (OR 2.7), endocrine diseases (OR 2.3), and other morbidity (OR 1.4), compared with women of normal weight. A high weight gain (>15 kg) during pregnancy was associated to later risk of overweight (OR 2.0) and obesity (OR 2.2), but not diabetes, cardiac disease, or endocrine diseases. A positive association was found between low weight gain and the risk of developing psychiatric disorders (OR 1.6).

**Conclusions:**

A high BMI at start of pregnancy significantly increased the risk of several diseases later in life. However, a high weight gain during pregnancy was only significant for future overweight and obesity. These findings have implications for both pregestational intervention and post gestational follow up of obese and overweight women.

## Background

Overweight and obesity are increasing worldwide and also becoming more common among young people. In population-based birth cohorts in the UK, trends towards higher BMI values at younger ages have been demonstrated [1], which means earlier exposure to risk and earlier development of complications. The prevalence of overweight and obesity in people of childbearing age in Sweden is estimated to be around 40%. Adiposity promotes inflammation and increases the frequency of metabolic and cardiovascular diseases, and obesity is a well-described risk factor in elderly people. Even at a young age, obesity has a significant impact on mortality and morbidity. In a Swedish follow-up study of 700,000 18-year-old men, there was a 2.2-fold increase in total mortality rate 45–60 years later among young obese men compared with men of normal weight. The causes of death were predominantly coronary heart disease, stroke and cancer [2]. In obese and overweight women, pregnancies and deliveries are more often associated with hypertension, preeclampsia and gestational diabetes, and higher frequency of infants that are large for gestational age [3]. These immediate complications of obesity are well known, but the long-term consequences for the health of these women are understudied.

Obesity during pregnancy can be due to pre-existing overweight or obesity, or due to an excessive gestational weight gain (GWG) during pregnancy, or a combination of both. Both high pregestational BMI and high GWG during pregnancy are risk factors for gestational complications and macrosomia [4].

A high GWG is associated with greater risk of perinatal morbidity and greater risk of high birth weight of the infant [5,6]. A high GWG is also associated with weight retention after delivery [7,8], which is more pronounced in women with pregestational overweight and obesity and predisposes to an even worse situation in subsequent pregnancies.

The Institute of Medicine (IOM) has given recommendations on appropriate GWG in different BMI intervals [9]. These goals for a limited weight gain during pregnancy in relation to overweight at the start of pregnancy constitute an attempt to avoid or at least diminish maternal health problems after delivery. A better maternal outcome has also been demonstrated in women with type 2 diabetes who followed these recommendations. Similarly positive results of a limited GWG were seen for obese women [10,11], and in another study for both women of normal weight and obese women [12].

The aim of this study was to analyse how maternal BMI at the start of pregnancy and maternal weight gain during pregnancy impact the risk of developing different diseases later in life.

## Research Design and Methods

The study was approved by the Ethical Review Board at Lund University (LU 632–03, 849/2005 and 94/2011). This was a retrospective study, using a population-based cohort of 23,524 women from southern Sweden. The cohort was originally designed for the study of melanoma and every fourth woman in the Population Register was asked to participate[13]. The women were asked to complete a questionnaire when they were 35–65 years old. From the questionnaires, we collected information on the weight and height of the women and any current diseases. The questions were constructed as yes or no answering to if the woman has diabetes, cardiac disease, endocrine disease, any psychiatric disease, has had stroke or has any other disease not listed. These data were self-reported by the women. We matched this cohort with the Swedish Medical Birth Register (SMBR) to identify all women with pregnancies resulting in delivery of an infant. Almost all births in Sweden are registered in the SMBR, with an approximate dropout rate of only 0.5–3%. The register contains data regarding the woman, the pregnancy, and the delivery, as well as postnatal data regarding the infant [14]. We found 13,608 women in our cohort who had at least one registered delivery in the SMBR between 1973 when the register was started and 1991. Only data from the first registered pregnancy for each woman were included in the study. From the SMBR, we collected information regarding the women’s weight and height at the beginning of the pregnancy, and weight gain during the pregnancy. Weight and height registration was often omitted in the SMBR at the beginning of the pregnancy. In some cases early pregnancy weight was calculated from weight at delivery minus weight gain during pregnancy. BMI could be obtained for 3,539 women at the beginning of pregnancy. In SMBR, the reporting of weight and weight gain varied over time since 1973. Data regarding weight gain in our study was, in some cases, provided directly from SMBR and in other cases it was calculated from last obtained weight prior to delivery minus early pregnancy weight. The health care professionals, and mostly midwifes register data in SMBR and these data are based on objective measurements using scales. The first registered weight during pregnancy, which was from around the 10^th^-12^th^ gestational week, was defined as pregestational weight in our study and is presented as early pregnancy weight in the following text. The women were divided into groups based on their first registered BMI (≤25 and >25; n = 3098 and n = 441, respectively) and based on weight gain during pregnancy (≤15 kg and >15 kg; n = 2768 and n = 1661, respectively).

The data in the questionnaires regarding medical history, diseases and medication were compared with data from the women’s first registered pregnancy in the SMBR. The follow-up time from delivery to questionnaire ranged from 10–17 years. The observed mean GWG in the whole cohort was 14.8 kg and we therefore in this study defined a weight gain of 15 kg or more as an excessive weight gain. Early pregnancy BMI >25 and weight gain >15 kg during pregnancy were assessed separately as well as in a multivariate analysis as potential risk factors for future diseases.

Statistical analyses were performed using the SPSS version 22 statistical software for PC, (SPSS Inc, Chicago, Illinois). Means and standard deviations are reported for normally distributed data such as age and GWG. Medians and ranges are reported for BMI groups. Frequencies in percent are reported for categorical variables such as diabetes, obesity and cardiac disease. Student’s T-test was used to compare age, early gestational weight and weight gain between women with available BMI and those without BMI to control for bias. For comparison of weight, GWG and BMI between the different BMI groups, an ANOVA test was used. To measure the separate associations between overweight and obesity for future outcomes, odds ratios (ORs) were calculated using Chi 2 tests. Overweight and obesity were handled as categorical values. In a binary logistic regression analysis both early pregnancy BMI and GWG were combined to evaluate the risk of future diseases. In these models BMI and GWG were used as continuous variables. To evaluate the impact of GWG in different BMI groups, separate regression analyses were done. P-values <0.05 were considered statistically significant.

## Results

We found 13,608 women from the southern part of Sweden who were eligible for the study. BMI in early pregnancy was only available for 3,539 women. Mean age of women with known BMI early in first pregnancy was 27.0±4.4 (n = 3539) compared to 26.8±5.1 years (n = 10,069); p = 0.1 in women without known BMI. The mean height was 167±5.7 cm (n = 2814) vs. 166±5.8 cm (n = 8053); p<0.001, mean weight was 60.9±8.8 (n = 3539) vs. 60.1±8.2 kg (n = 139); p = 0.3, and mean GWG was 14.4±4.6 (n = 3537) vs. 14.8±4.5 kg (n = 890); p = 0.015). The mean BMI for all women at follow up was 24.6±4.1 kg/m^2^ (n = 10,632), and women with known BMI in early pregnancy had BMI 24.3±4.1 (n = 2750) at follow up while women without known BMI during pregnancy had BMI 24.7±4.0 (n = 7882); p<0.001 at follow up. Weight gain during pregnancy and BMI at follow up, in relation to BMI early in pregnancy is presented in Table 1.

**Table 1:** Characteristics of women by BMI in early pregnancy.

	BMI <20	BMI 20–25	BMI 25–30	BMI >30	P-value
**Mean age at first pregnancy (Yrs±SD) *(n = 3*,*539)***	26.7±4.3	27.1±4.4	26.7±4.8	28.8±6.1	0.002
	*(903)*	*(2195)*	*(384)*	*(384)*	
**Early pregnancy weight (Kg) *(n = 3*,*539)***	52.4±4.5	61.3±5.5	74.7±5.7	87.1±6.5	<0.001
	*(903)*	*(2195)*	*(384)*	*(57)*	
			
**Median BMI at start of pregnancy (Kg/m^2^) *(n = 2*,*539)***	19.1	21.8	26.5	31.6	<0.001
	13.1–20.0	20.0–25.0	4.8–25.0	30.1–40.5	
	*(903)*	*(2195)*	*(384)*	*(57)*	
**Mean maternal weight gain (Kg) *(n = 3*,*537)***	13.8±4.1	14.8±4.6	14.7±5.2	8.9±4.8	<0.001
	*(903)*	*(2195)*	*(384)*	*(55)*	
**Mean BMI at follow up 10–17 yrs after pregnancy (Kg/m^2^) (n = 2,750)**	21.6±2.3	*24*.*3±3*.*1*	29.7±5.0	33.1±7.0	<0.001
	(710)	*(1700)*	(293)	(47)	

**Fig 1:**
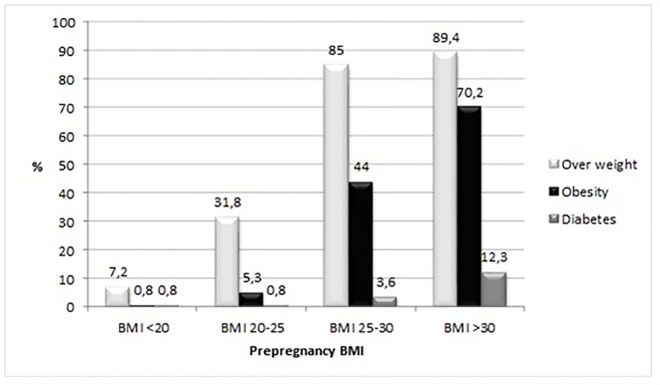
Frequency of different diseases 10–17 years after pregnancy by BMI group.

At follow up, 10–17 years after pregnancy, 219 (1.6%) of the women had been diagnosed with diabetes. In the highest BMI group with BMI >30, 12.3% of the women had been diagnosed with diabetes (Fig 1). In the study group as a whole, 37.0% of the women were overweight or obese at follow up, and 9.4% were obese (BMI >30). Among women with BMI <20 at the beginning of their first pregnancy, only 7.2% were overweight and 0.8% obese at follow up. With higher BMI at the start of pregnancy, the frequency of both overweight and obesity at follow-up also increased. The proportion of overweight was 85% and obesity 44% in women with BMI 25–30 at the start of pregnancy. Most, but not all women with BMI above 30 at the start of pregnancy were obese at follow up (70.2%) but an even higher proportion were overweight (89.4%) (Fig 1).

There were 356/10818 (3.3%) women with cardiac diseases, 770/10867 (7.1%) with endocrine diseases, 46/10919 (0.4%) women with stroke, and 302/10890 (2.8%) women with psychiatric diseases in the whole group of women at follow up. For women with known BMI in early pregnancy and included in our risk estimations the frequencies of these diseases are given in Table 2 and Table 3. There was a positive association between BMI >25 and the risk of developing obesity (OR 21.9; p-value <0.001), diabetes (OR 6.4; p-value <0.001), cardiac disease (OR 2.7; p-value <0.001), endocrine diseases (OR 2.3; p-value <0.001) and overall/other morbidity (OR 1.4; p-value = 0.013) (Table 2).

**Table 2:** High BMI (>25) at start of pregnancy and the risk of different diseases 10–17 years after pregnancy.

	Affected numbers/ Total observations (%)	OR (BMI >25 vs.≤25)	95% CI	P-value
**Obesity (BMI ≥30)**	258/2750 (9.4%)	21.9	16.3–29.5	<0.001
**Diabetes**	45/3537 (1.3%)	6.4	3.5–11.6	<0.001
**Cardiac disease**	55/2816 (2.0%)	2.7	1.5–4.9	0.001
**Stroke**	7/2835 (0.2%)	1.2	0.2–9.6	NS
**Endocrine disease**	149/2817 (5.3)	2.3	1.5–3.4	<0.001
**Psychiatric disease**	82/2822 (2.9%)	1.6	0.9–2.8	NS
**Other morbidity**	809/2723 (29.7%)	1.4	1.1–1.7	0.013

**Table 3:** High weight gain and the risk of different diseases 10–17 years after pregnancy.

	Affected numbers/Total observations (%)	OR >15kg vs. ≤15kg	95% CI	P-value
**Overweight (BMI 25–30)**	1089/3448 (31.6%)	2.0	1.7–2.3	<0.001
**Obesity (BMI ≥30)**	297/3448 (8.6%)	2.2	1.7–2.8	<0.001
**Diabetes**	54/4429 (1.2%)	0.6	0.4–1.2	NS
**Cardiac disease**	66/3524 (1.9%)	0.7	0.4–1.3	NS
**Stroke**	8/3541 (0.2%)	2.9	0.7–12.0	NS
**Endocrine disease**	189/3522 (5.4%)	0.9	0.7–1.3	NS
**Psychiatric disease**	102/3530 (2.9%)	0.6	0.4–0.9	0.03
**Other disease**	999/3405 (29.3%)	1.0	0.9–1.2	NS

There was a positive association between low maternal weight gain and the risk of developing psychiatric diseases (OR 1.6; p<0.001). There was, however, no significant association between high weight gain and the risk of developing diabetes, cardiac diseases, or endocrine diseases 10–17 years after delivery (Table 3).

Both early gestational weight and weigh gain were included in a multivariate model as risk factors for diabetes, overweight and obesity (Table 4). In combined analyses with early gestational BMI and GWG as continuous variables, only early gestational BMI was of significance for later development of diabetes, while both variables were associated with future overweight and obesity (Table 4).

**Table 4:** Early gestational BMI and gestational weight gain as risk factors for diabetes, overweight and obesity in a multivariate analysis.

Model 1 –risk for diabetes n = 3537	OR	95% CI	P-value
**Early gestational BMI (kg/m**^**2**^**)**	1.1	1.0–1.1	0.043
**Weight gain (kg)**	1.0	0.9–1.0	NS
**Maternal age**	1.0	1.0–1.1	NS
**Model 2 –risk for overweight n = 2749**			
**Early gestational BMI (kg/m**^**2**^**)**	1.8	1.71–1.89	<0.001
**Weight gain (kg)**	1.1	1.06–1.11	<0.001
**Maternal age**	1.0	0.96–1.01	NS
**Model 3 –risk for obesity n = 2749**			
**Early gestational BMI (kg/m**^**2**^**)**	1.8	1.68–1.89	<0.001
**Weight gain (kg)**	1.14	1.11–1.18	<0.001
**Maternal age**	0.95	0.91–0.94	0.005

**Fig 2:**
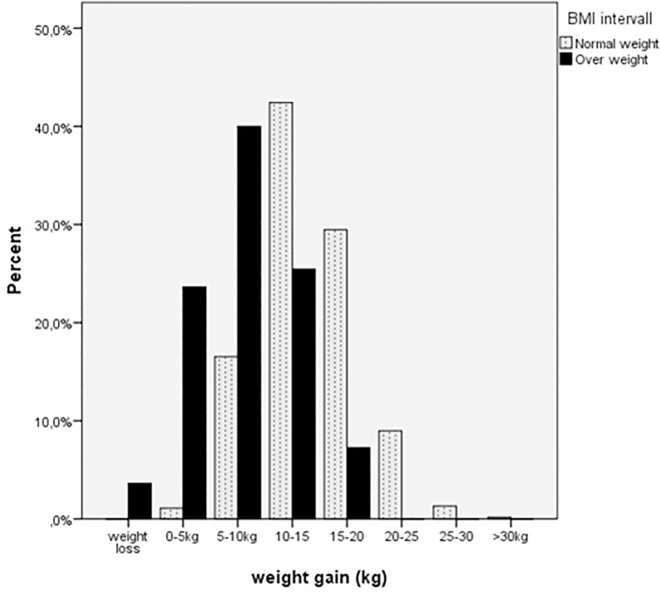
Weight gain in different BMI groups Women who were overweight (BMI>25) before pregnancy had a lower weight gain compared to normal weight (BMI≤25) women.

The women who were overweight or obese had a lower weight gain during pregnancy than the women who were of normal weight or underweight (Fig 2). In particular, the women who had an early gestational BMI >30 had a significantly lower weight gain compared to all other women. The mean weight gain in this group was 8.9 kg and was just within the recommendations in the IOM guidelines, whereas the mean GWG in the group of women who were overweight (BMI 25–30) was well above the IOM recommendations. The weight gains in women who were of normal weight or underweight were within the IOM recommendations (Table 1).

We also did regression analyses for separate BMI groups (BMI <25, BMI 25–30 and BMI >30) to examine if GWG had different impact depending on early pregnancy BMI. A high GWG increased the risk of overweight later in life among normal weight (n = 1700, 95% CI 1.05–1.11, p<0.001) and overweight women (n = 293, 95% CI 1.08–1.25, p<0.001). There was an increased risk of obesity later in life for women with normal weight or overweight with a high GWG (n = 1923), 95% CI 1.12–1.23 and 1.05–1.16; p<0.001 for both). A high GWG only significantly increased the risk of diabetes in the group of women who were obese at the beginning of pregnancy (n = 55), 95%CI 1.04–1.9; p = 0.03). There was no increased risk for cardiac diseases, stroke or endocrine diseases with increasing GWG in any BMI group. A high GWG decreased the risk of psychiatric diseases among women who were overweight in early pregnancy (n = 307), 95%CI 0.77–0.99; p = 0.03), but we did not observe a significant difference in other BMI groups.

## Discussion

In this large retrospective cohort study, we found a six-fold increase in the risk of developing diabetes, more than a two-fold increase in risk for cardiac and endocrine diseases, and 1.4-fold increase in risk for all other diseases, 10–17 years after first registered delivery in women with BMI above 25 at start of pregnancy. Not surprisingly, there was a huge increase in the risk of obesity, defined as a BMI >30, with an OR as high as 21.9 if the woman was already overweight or obese before pregnancy.

Obesity during pregnancy may be either due to a high preconception weight in the woman or an excessive weight gain during pregnancy. In general, a low GWG among obese women during pregnancy is supposed to be beneficial and acknowledged to reduce the risk for complications, such as perinatal morbidity, and the risk of Large for gestational age (LGA)-infants (10, 11). With the purpose to improve maternal and child health after delivery the IOM has published recommendations for GWG (5–9 kg for obese, 7–11.5 kg for overweight, and 11.5–16 for normal weight women), to try to reduce excessive weight gain in women who are already overweight. It has even been suggested that weight loss during pregnancy could be beneficial, but a systematic review has shown that weight loss is associated with an increased risk of small for gestational (SGA) infants and possibly also preterm deliveries [15]. In our study, the women with obesity (BMI>30) before pregnancy had the lowest weight gain (mean 8.9 kg) of all BMI groups, which was within the IOM recommendations for obese women. These pregnancies predate the IOM recommendations and to our knowledge, the women were not given any special advice regarding weight gain during pregnancy. This could in part explain our observed lower risk for later metabolic diseases, due to high GWG compared to the risk of high BMI in early pregnancy. On the other hand, the overweight women had a mean gestational weight gain well above the IOM recommendations.

In this study we have tried to separate high early gestational weight and high GWG as risk factors for future disease. The risk for future diabetes among overweight pregnant women was increased by as much as six times in our study compared with women of normal weight. The majority of the women in our study were not screened for gestational diabetes during pregnancy and the frequency of reported gestational diabetes mellitus (GDM) in the SMBR was very low. We have previously characterised the pregnancies of the women who later developed diabetes in this cohort and found that compared with women who did not develop diabetes, they had a higher weight at start of pregnancy (69.2 vs. 63.2 kg; p<0.001), gave birth to larger infants (3,602gvs. 3,507g; p<0.001) despite a shorter gestational length and had a higher frequency of caesarean sections, all typical features of GDM [16]. Using an oral glucose tolerance test as a general screening procedure during pregnancy would probably identify these women with GDM, and intervention would possibly delay or prevent the development of diabetes.

The impact of a high GWG on the future risk for diseases in the mother has been studied before. In our study, high weight gain did not appear to be a risk factor for the development of diabetes unless the women were obese in early pregnancy. In contrast, in an Australian study, the risk of diabetes 21 years after pregnancy was 1.47 times higher among the women with an excessive weight gain during pregnancy compared to women with a normal weight gain [17]. However, similar to our observations, the risk was only increased for women who had a BMI above normal before pregnancy, and early pregnancy weight could therefore play a more important role.

In our population, we found a 2.7-fold increase in risk for cardiac events in women with a BMI over 25 before pregnancy. This is comparable with a recent Danish study of obese women that showed a 2.6-fold increase in risk for myocardial infarctions and a 1.9-fold increase in risk for stroke after 4.5 years of follow-up [18]. A similar increase in risk for men has been described in a prospective study of more than 3000 men between 18 and 30 years old, where BMI was found to be directly associated with myocardial systolic dysfunction after 25 years of follow-up [19]. In 12,850 men examined before military service in Denmark and followed for 36 years, obesity was associated with an increased risk of myocardial infarction (OR 2.9), unstable angina (OR 5.5) and congestive heart failure (OR 6.7) [20].

Endocrine disorders, without further separation into thyroid, adrenal or other hormonal dysfunction, were reported at a higher frequency in mothers with a BMI above 25 compared with those with a BMI below 25. The possible relationship between thyroid diseases and obesity has been debated [21,22], as well as the relationship between obesity and the hypothalamic-pituitary-adrenal axis [23]. We also found an increase in overall morbidity in diseases previously not mentioned. Previous reports indicate, for example, an association of obesity with certain types of cancer [24] as well as musculoskeletal pain syndromes and obesity seems to have an impact on the risk of a variety of disorders.

The frequency of overweight and obesity is increasing in the general population. Excessive weight gain in women often starts during or after pregnancy. Some studies have shown that a high GWG seems to have a greater impact on the risk of obesity later in life than early pregnancy BMI itself [25,26]. In our study we found an OR of 19 and 22 for future overweight and obesity respectively, for women with BMI >25 at the start of pregnancy compared with women with BMI ≤25, and a significant but smaller increase in risk associated with high GWG. Many of the studies proposing GWG as a risk factor have defined a high GWG in relation to the recommendations of the IOM, where a lower weight gain is recommended for overweight and obese women than for lean women, whereas we explored the absolute weight gain. This could partly explain the diverging results.

Depression and other psychiatric disorders have been studied in relation to weight and weight gain during pregnancy, but the results are inconclusive. In a prospective cohort study of Hispanic women, Ertel et al showed a strong negative association between prepregnancy BMI and depressive symptoms that remained after controlling for potential confounders, but no association between depression and gestational weight gain [27]. In contrast, another study indicated that obese women who got pregnant were significantly more likely to experience both antenatal and postpartum depression symptoms compared to women of normal weight [28].

In our study, we observed an increased risk of psychiatric diseases among women with a low gestational weight gain. These apparently inconsistent results may be explained by the findings in a Danish study, where both increases and decreases in weight in relation to previous weight were associated with depression and anxiety disorders [29]. Weight and its relation to depressive disorders in the pregnant woman merits further investigation.

A weakness of our study is that in this cohort we had a lot of missing data on weight or height, which made it impossible to calculate BMI. We adhered to testing associations against BMI rather than weight, in order to get the best approximations for overweight and obesity. However, there is no reason to believe that missing data are anything but random since age differed only with 0.2 yrs while initial weight and weight gain were equal in both groups. Loss of data was therefore not considered to give any bias to the results. We used the first registered weight during pregnancy as an approximation for prepregnancy weight. Since the health care provider did these measurements we think that the reliability was higher than from self-reported data. Weight gain during first trimester was disregarded since most weight gain occurs after first trimester. Weight measurements recorded early in pregnancy are considered sufficiently accurate for substitution when prepregnancy weight is unavailable [30]. Furthermore, the diseases at follow up were self-reported, and the quality of these data was therefore not as good as it would have been if they had been based on professional records or pharmacy registers.

Strength of our study is that the cohort was population-based, with every fourth woman in the Population Register being asked to participate. The initial purpose was to act as a control population in a study of malignant melanoma, and any bias towards metabolic diseases is therefore not to be expected. The data from the Swedish Medical Birth Register is highly valid since the ascertainment rate is almost 100%.

Obesity is increasing in the population as a whole, and especially in young subjects. This will have implications not only for obstetricians but also for the whole health care system. Cardiovascular diseases, which are closely associated with obesity, are one of the leading causes of death and are responsible for high costs for hospitalisation and care.

For women who become pregnant, being overweight not only implies a risk to their own future health but also to the health of their children. The highest risk for overweight in children aged 3–6 years was seen when their mothers were overweight or obese before pregnancy and gained excess weight during pregnancy [31]. Furthermore, it has been shown that obesity in the parents more than doubles the risk of adulthood obesity in the offspring [32]. We will therefore face an even larger problem with obesity in the coming generation than we are facing today. A risk assessment based on childhood BMI, mother’s BMI and family income is superior to risk assessment with BMI in combination with biomarkers and genetic markers in predicting adulthood obesity [33] and it is known that obesity in pregnant women as well as in the general population is much more common in low income groups [34]. The positive aspect of this is that environmental factors are possible to change, in contrast to genes.

The problem of obesity during pregnancy is well known in obstetric and maternity care units but it is not an easy problem to solve. Normal weight before pregnancy should be aspired to, and an increased awareness is necessary in the general population about the risk for poor perinatal outcome that is associated with obesity [35]. The mother’s lifestyle during and after pregnancy is crucial for her future health [36], but interventions addressing diet and physical activity are not sufficient to prevent a poor outcome [37]. Promotion of physical activity and prevention of declining activity during pregnancy seems to be a promising approach [35] as also concluded in a Cochrane analysis [38]. In the US, a home-based service for pregnant women with BMI >30 has been started but has not yet been evaluated [39]. Bariatric surgery and gastric by-pass in young women are invasive procedures intended to establish normal weight before pregnancy, but they increase the risk of delivering infants that are small for gestational age [40]. Early intervention and increased focus on socioeconomic factors and ethnicity seem necessary [41].

### Conclusion

A high BMI at the start of pregnancy significantly increases the risk of several diseases in later life, including diabetes and cardiac disease. A high weight gain during pregnancy leads to overweight and obesity but does not appear, in our study, to have any implications for metabolic diseases later in life.
